# Introgression despite minimal hybridization: mating system modulates phenotypic associations with introgression in *Clarkia*

**DOI:** 10.1111/nph.71113

**Published:** 2026-03-27

**Authors:** Shelley A. Sianta, Brooke R. Kern, Amal Suri, Yaniv Brandvain, David A. Moeller

**Affiliations:** ^1^ Department of Plant and Microbial Biology University of Minnesota 1479 Gortner Avenue St. Paul MN 55108 USA; ^2^ Department of Integrative Biology The University of Texas at Austin 2415 Speedway Austin TX 78712 USA

**Keywords:** admixture, chloroplast capture, flower color, hybrid zone, outcrossing and selfing, reproductive isolation, spatial genetic structure, speciation

## Abstract

- Secondary contact between incipient species provides the opportunity to understand how trait divergence restricts gene flow. While mating system transitions are particularly common and involve a suite of phenotypic changes, the extent to which these changes influence gene flow during speciation is poorly understood.
- Using 410 genomes, we quantified introgression between selfing‐outcrossing sister *Clarkia* taxa in four independent contact zones. We used common garden and geospatial data to determine the frequency of early generation hybrids and test for associations between individual admixture proportions and phenotypic and spatial variation within contact zones.
- We found substantial admixture across contact zones, but no early generation hybrids. Introgression was consistently asymmetric, from the selfer to outcrosser. Patterns of chloroplast capture suggest that F1 hybrids often form on the selfer. Although the extent of admixture varied among contact zones, only the selfer displayed spatially structured and phenotypic associations with introgression. Secondary sexual traits that minimize pollinator visitation in the selfer (e.g. flower color) may reduce admixture and promote asymmetric introgression.
- Despite recent divergence and evidence of historical introgression, premating isolation minimizes hybridization. Mating system consistently modulates the direction of introgression and phenotypic variation appears to contribute to the dynamics of backcrossing following F1 formation.

## Introduction

Understanding the earliest stages of the speciation process is important for determining which trait changes most strongly affect reproductive isolation and minimize introgression. In flowering plants, pollinator shifts are well known to cause the rapid evolution of reproductive isolation (Grant, 1949; Kay & Sargent, 2009; Van der Niet *et al*., 2014). At the same time, many clades exhibit conservatism of pollination systems (Ollerton *et al*., 2019; Kriebel *et al*., 2023; Martín‐Hernanz *et al*., 2023), suggesting that other factors (e.g. local adaptation) likely contribute to reproductive isolation in the early stages of divergence. Mating system transitions are common in the evolutionary history of flowering plants (Stebbins, 1974) but our understanding of their contribution to plant speciation remains incomplete.

Shifts from outcrossing to selfing are often found toward the tips of phylogenetic trees (Schoen *et al*., 1997; Takebayashi & Morrell, 2001; Igic & Busch, 2013), suggesting that many mating system transitions are recent. Mating system shifts often occur when selfing taxa “bud off” from outcrossing taxa at the periphery of the geographic range, eventually leading to sister taxa with parapatric ranges (Gottlieb, 1973; Pettengill & Moeller, 2012a; Willi *et al*., 2022). In this divergence history, an initial period of allopatry may be short lived and incipient sister taxa with divergent mating systems are unlikely to remain distinct following secondary contact without some degree of reproductive isolation. Experimental studies show that reproductive isolation can rapidly evolve in concert with mating system divergence, primarily through traits that mediate premating isolation (Martin & Willis, 2007; Koelling & Mauricio, 2010; Briscoe Runquist *et al*., 2014; Brys *et al*., 2014; Rifkin *et al*., 2023). However, few studies have determined whether insights from experimental studies of reproductive isolation translate to limited hybridization and introgression in natural contact zones (but see Martin & Willis, 2007; Brys *et al*., 2014; Farnitano *et al*., 2025).

Population genomic studies of outcrosser–selfer taxon pairs have revealed an emergent pattern of asymmetry in introgression (Pickup *et al*., 2019). Introgression occurs primarily from the derived selfing taxon to the ancestral outcrossing taxon (Ruhsam *et al*., 2011, 2013; Pettengill & Moeller, 2012a; Brandvain *et al*., 2014; Kenney & Sweigart, 2016; Rifkin *et al*., 2019; Sianta *et al*., 2024; Farnitano *et al*., 2025). While this pattern appears consistent (Pickup *et al*., 2019), the mechanism by which asymmetric introgression occurs remains unclear. Asymmetric introgression can occur when F1 hybrids differentially backcross to one parent species. Even when rates of backcrossing to both parent species are similar, asymmetric introgression may occur when selection against admixed ancestry is stronger in one parental background. Little is understood about either possible mechanism that could account for similarities among selfer–outcrosser pairs (but see Ruhsam *et al*., 2013).

The extent and direction of gene flow between plants of differing mating systems could be influenced by floral trait changes that directly increase autonomous self‐fertilization and floral trait changes that accompany selfing but are not required for it. Traits that directly affect selfing (e.g. reduced dichogamy and herkogamy) are also thought to evolve more quickly in the early stages of the evolution of selfing (e.g. Vallejo‐Marín & Barrett, 2009; Kalisz *et al*., 2012; Gervasi & Schiestl, 2017). Such traits are known to minimize the probability that hybrids form on selfers (Fishman & Wyatt, 1999; Smith & Rausher, 2007; Brys *et al*., 2016) and could influence subsequent patterns of introgression. For example, if selfing minimizes hybrid formation on selfer genotypes, then it follows that F1s (with intermediate traits) are more likely to backcross primarily to the outcrosser. Floral changes indirectly associated with selfing (e.g. reduced flower size, pollen, and nectar production) can reduce pollinator visitation and male fitness (Moeller & Geber, 2005; Goodwillie *et al*., 2010; Briscoe Runquist *et al*., 2017) and can therefore influence both F1 formation and subsequent patterns of backcrossing and introgression. Despite these plausible arguments, we are not aware of studies that have determined whether traits that directly mediate mating system vs those that mediate pollinator interactions are more important for influencing the degree of introgression between outcrossers and selfers.

Differential introgression could also be influenced by niche divergence, which often accompanies mating system transitions (Grant & Kalisz, 2019). For example, water availability drives spatial partitioning of co‐occurring *Mimulus guttatus* (outcrosser) and *M. nasutus* (selfer) along creek beds (Kiang & Hamrick, 1978; Farnitano *et al*., 2025). Hybrid zone models suggest that hybridization and admixture should be a function of distance to heterospecific individuals, reflecting the balance of dispersal and selection against admixed individuals in parental microhabitats (Moore, 1977; Barton & Hewitt, 1985). This spatial segregation associated with mating system divergence can influence gene flow in various ways. Spatial segregation can influence how pollinators mediate interspecific gene flow (Wesselingh & Arnold, 2000; Campbell *et al*., 2002), as studies of pollinator‐mediated pollen dispersal suggest that pollen receipt declines sharply with distance from the pollen donor (Schaal, 1980; Waser, 1988; Santa‐Martinez *et al*., 2021). Following hybrid formation, distance between plants may indirectly influence introgression because selection against admixed individuals is often stronger with distance from the ecotone between habitats (Milne *et al*., 2003; Campbell, 2004; Kimball *et al*., 2008; Abbott, 2017; DiVittorio *et al*., 2020; Massatti *et al*., 2025). Mating system may further modulate the spread of admixture throughout a population given differences in neighborhood size of outcrossers and selfers (Levin & Kerster, 1971).

Understanding the importance of mating system to introgression dynamics requires examination of the repeatability of introgression dynamics across independent contact zones within systems (Harrison & Larson, 2016; Langdon *et al*., 2024; Łabiszak *et al*., 2025). Geographic variation in introgression can reflect variation in the time period over which diverging lineages have been in secondary sympatry (van Riemsdijk *et al*., 2023). Alternatively, environmental variation can influence introgression by affecting the expression of traits that confer premating isolation and/or the nature of selection on admixed ancestry (e.g. Moran *et al*., 2025). For plant mating systems, similar patterns of introgression between outcrossers and selfers have been detected across systems. However, most of those studies have sampled either intensively within one contact zone (Kenney & Sweigart, 2016) or broadly across a large spatial scale with limited sampling within each contact zone (Brandvain *et al*., 2014; Rifkin *et al*., 2019; Sianta *et al*., 2024). These contrasting approaches are valuable for many questions but limit the capacity to evaluate the consistency of introgression dynamics across independent contact zones (but see Farnitano *et al*., 2025). Importantly, understanding introgression dynamics in any given contact zone requires sufficient sampling to be able to detect hybrid classes that are rare. Repeatable patterns of asymmetric introgression and trait mediation of admixture across contact zones would suggest that history or environment have minimal effects on reproductive isolation. By contrast, wide variation among contact zones would suggest that the contribution of mating system to introgression dynamics is context dependent.

**Fig. 1:**
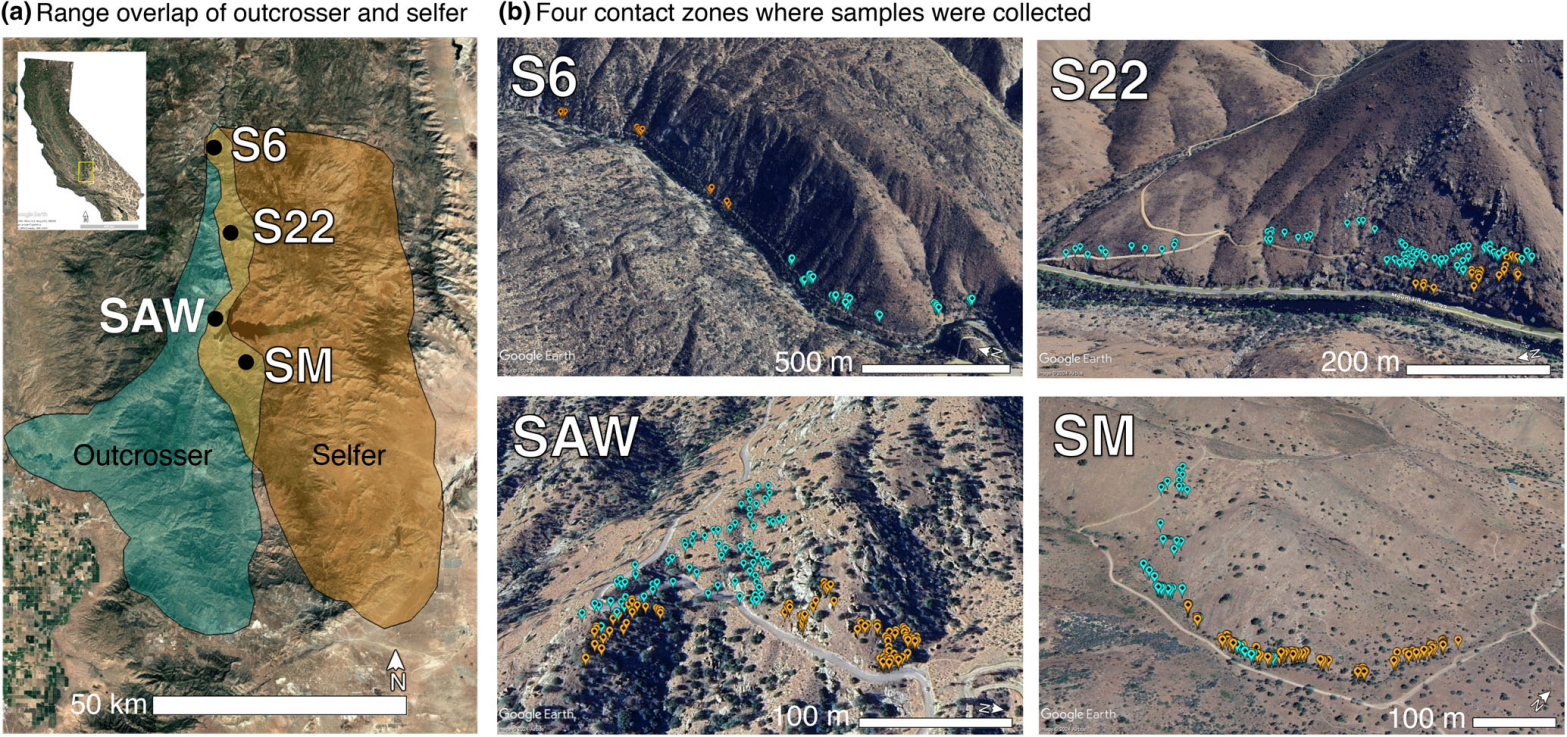
*Clarkia xantiana* ssp. *xantiana* (outcrosser) and ssp. *parviflora* (selfer) have parapatric distributions across broad and narrow spatial scales. (a) Range map of the outcrosser (blue) and selfer (orange), with regions of overlap in gold hue. The yellow box on the inset map of California represents the section of California in (a). (b) Individuals sampled at the four contact zones used in this study. Outcrosser individuals are in blue, selfer individuals are in orange. Maps were generated in Google Earth Pro. S6, Site 6; S22, Site 22; SAW, Sawmill Road; SM, Squirrel Mountain.

Here, we examined the influence of mating system on the magnitude and direction of introgression between two sister taxa in the genus *Clarkia*. The primarily selfing *Clarkia xantiana* ssp. *parviflora* (hereafter ‘selfer’) diverged recently (*c*. 56–65 kya) from the primarily outcrossing *C. xantiana* ssp. *xantiana* (hereafter ‘outcrosser’; Pettengill & Moeller, 2012b; Sianta *et al*., 2024). The taxa initially diverged in allopatry but now frequently co‐occur in discrete contact zones throughout a region of secondary sympatry (Fig. 1a; Pettengill & Moeller, 2012a). While premating isolation is strong (Briscoe Runquist *et al*., 2014), previous work has shown that introgression is ongoing and asymmetric (from selfer to outcrosser; Pettengill & Moeller, 2012b; Sianta *et al*., 2024). Within contact zones, the taxa are spatially partitioned (Fig. 1b), likely across microhabitats due to differences in physiology and life history (Eckhart *et al*., 2004; Burnette & Eckhart, 2021). Although both taxa are self‐compatible, mating system differences are caused by divergence in floral traits, which also vary substantially within each taxon (Runions & Geber, 2000; Moeller, 2006).

Our study focused on examining the mechanisms that cause asymmetric introgression, the extent to which phenotypic variation is associated with introgression, and the repeatability of introgression dynamics across contact zones. We densely sampled four contact zones (with georeferenced individuals) that span substantial environmental variation across the zone of secondary sympatry. We used genotyping‐by‐sequencing to quantify the frequency of early‐generation hybrids and the extent (and direction) of introgression. We used common garden experiments to quantify variation in traits that previous experiments have suggested most strongly cause premating isolation. We focused on the following questions:

1. What is the frequency of F1 hybrid formation? We evaluated this question using tests of modality for genomic and phenotypic variation within contact zones and triangle plots of genomic variation (quantifying the frequency of early generation hybrid classes).
2. Which taxon do F1s most often form on and what is the mechanism of asymmetric introgression? We evaluated this question by examining the maternally inherited chloroplast genome of individuals and the extent of chloroplast capture in each taxon.
3. What is the relationship between phenotypic variation and the degree of introgression between taxa? These relationships could indicate a role for mating system traits in modulating introgression and/or the effect of historic introgression on floral phenotypes.
4. What is the role of niche divergence in modulating the degree of admixture between taxa? We tested the hypothesis that admixture was greater for individuals closer to the heterospecific taxon.

## Materials and Methods

### Study system

*Clarkia xantiana* A. Gray (Onagraceae) is an annual herb endemic to the southern Sierra Nevada and Transverse Ranges of California, USA (Moore & Lewis, 1965; Eckhart & Geber, 1999). The two focal taxa are currently described as subspecies of *C. xantiana*. However, they may be considered distinct species because they are phylogenetically, genomically, and morphologically distinct as well as partially cross‐incompatible (Pettengill & Moeller, 2012b; Briscoe Runquist *et al*., 2014; Sianta *et al*., 2024). Past phylogenetic, demographic modeling, and population genomic work demonstrated that initial divergence occurred in allopatry and that sympatry is a result of range shifts north and to higher elevations (Pettengill & Moeller, 2012a; Sianta *et al*., 2024). The two taxa have parapatric distributions along a west‐to‐east precipitation gradient that spans the southern Sierra Nevada (Eckhart & Geber, 1999; Geber & Eckhart, 2005). The outcrosser occurs in more mesic oak‐pine woodlands of the western foothills, whereas the selfer occurs in xeric scrub habitats of the eastern foothills that extend to the Mojave Desert. The taxa are divergent in physiological and life history traits and drought adaptation strategies (Mazer *et al*., 2010; Burnette & Eckhart, 2021). Flowers of the outcrosser are pink, whereas those of the selfer may be pink or white. The white color morph is found throughout much of the taxon's range but not in every population (Eckhart & Geber, 1999). White has evolved independently from pink multiple times as evidenced by a phylogeny of selfer populations and crossing experiments (unpublished observations).

### Field sampling

In 2019, we collected seeds from three contact zones: Site 22 (S22), Sawmill Road (SAW), and Squirrel Mountain (SM) (Fig. 1b). In 2021, we collected from Site 6 (S6), the northernmost documented contact zone. These sites span 42 km of the region of secondary sympatry. Plants of both taxa were abundant in 2019 and 2021 during collections. We visited each site during flowering to haphazardly mark individuals across the full extent of each taxon's population. Individuals were typically spaced by > 3 m and chosen irrespective of size, vigor, or trait variation. The number of individuals per taxon was roughly proportional to the area covered by each population. Individuals were georeferenced at the time of fruit collection (Garmin GPSMAP 64st). In total, we collected 433 individuals: 75 from S6 (53 outcrossers, 22 selfers), 109 from S22 (78 outcrossers, 31 selfers), 153 from SAW (65 outcrossers, 88 selfers), and 96 from SM (23 outcrossers, 73 selfers).

### Common garden phenotyping

One seed per maternal plant was germinated and grown to flowering in the glasshouse to phenotype and collect leaf tissue for population genomics. We grew plants in conetainers (Stuewe and Sons; 174 ml) with potting soil (Sun Gro Sunshine Mix #1). We germinated seeds at 13°C/12 h days in growth chambers and grew seedlings under the same conditions for *c*. 4 wk. We moved seedlings to a glasshouse (*c*. 18–28°C; 14 h days with supplemental light for 6 h day^−1^). Phenotyping for SAW, S22, SM, and S6 occurred in fall 2019, winter 2020, winter 2020, and fall 2021, respectively. Leaf tissue was harvested before flowering and stored at −20°C until DNA extraction.

We phenotyped individuals in the glasshouse for traits that have been shown to distinguish the two taxa and are involved in mating system and/or reproductive isolation (Eckhart & Geber, 1999; Runions & Geber, 2000; Moeller, 2006). For each plant, we recorded flower color (pink or white), measured petal size (using ImageJ to quantify petal area; Schneider *et al*., 2012), herkogamy (distance in the nearest half millimeter between the receptive stigma and the nearest anther), protandry (time in the nearest half day between long anther dehiscence and stigma receptivity), and the day of first flower. Stigmas were considered receptive when the stigma surface was flat (Ruane *et al*., 2020). Floral traits were measured for two flowers on each plant; values from the two flowers were averaged for analyses. Day of first flower (hereafter, ‘flowering time’) was recorded and standardized by the earliest flowering individual in each contact zone.

### DNA sequencing and bioinformatics

DNA was extracted from leaf tissue with the CTAB method (Doyle & Doyle, 1987). The University of Minnesota Genomics Center (UMGC) double‐digested DNA with ApeKI and MboI enzymes and prepared libraries for multiplex sequencing with the Illumina Nextera Library Preparation Kit. In brief, Illumina Nextera adapters were ligated to DNA samples, CDI barcodes were added with 18 cycles of PCR, libraries were SPRI purified, quantified, and pooled, and fragments within 300–750 bp were selected for sequencing. Samples from S22, SAW, and SM (*n* = 377) were sequenced on two lanes of an Illumina NovaSeq 6000 (Illumina, CA, USA), resulting in a total of 1 840 880 108 150‐bp paired end reads with an average of 4 882 971 read‐pairs per sample. Reads covered an average of 19.8% of the genome. Samples from S6 (*n* = 75) were sequenced on one lane of an Illumina NovaSeq S1, resulting in 717 110 495 150‐bp paired end reads with an average of 9 561 473 read pairs per sample. Similar to the first run, an average of 21.9% of the genome was covered.

We removed adaptors with a custom script from UMGC (Garbe, 2019) and used Trimmomatic (v.0.33; Bolger *et al*., 2014) to remove bases at leading and trailing ends with quality scores below 10, 5‐base sliding windows with average quality scores below 20, and reads with < 75 remaining bases. An average of 93% of read pairs passed filtering at this stage. We mapped trimmed reads to the unmasked *C. xantiana* ssp. *parviflora* reference genome (Sianta *et al*., 2024) with BWA *mem* using default parameters (v.0.7.17; Li & Durbin, 2009). For each alignment, we used SAMtools (v.1.16.1; Danecek *et al*., 2021) to sort reads, fix mates, mark duplicate reads, and index the alignment; we used Picard (v.2.25.6; Broad Institute, 2019) to add read groups.

We called genotypes with BCFtools (v.1.16.1; Danecek *et al*., 2021) using the *mpileup* and *call* commands. We removed sites that had > 25% missing individuals, individuals with > 19% missing sites (*n* = 23), and scaffolds in < 5% and > 90% quantiles for average depth (3% of sites). After filtering, average depth across all individuals was 2.7×. We further identified and removed 1 kb windows that included 10 or more heterozygous sites for > 50% of selfer individuals. These regions with unusually high heterozygosity may include paralogs and/or represent poor alignments. In addition, we removed sites with low genotyping quality (QUAL < 30), sites with low mapping quality (MQ < 30), and sites with high sample depth (FMT/DP > 100×). The resulting VCF contained 16 294 899 sites, of which 2104 244 were biallelic single‐nucleotide polymorphisms (SNPs).

To determine the extent of chloroplast capture, we amplified a taxon‐specific chloroplast SNP. We used the whole‐genome‐sequence dataset from Sianta *et al*. (2024) to search for SNPs with fixed differences between allopatric populations of each taxon. We designed primers for three SNPs with the NCBI primer design tool (Ye *et al*., 2012) and chose one SNP that amplified well in trials. The chosen SNP (5′‐3′ primers: F: ACCGCCCGTTCAGATACATTT; R: AGGGTCTTCCTCTTTGAGGG) falls within the chloroplast genome that is embedded within the reference genome Super‐Scaffold_100056 (position 457 410 in reference sequence). We used PCR to amplify sequences (initial denaturation at 95°C for 1 min; 30 cycles of denaturation at 95°C for 20 s, annealing at 54°C for 30 s; extension at 68°C for 30 s; final extension at 68°C for 3 min) and sent PCR products for Sanger sequencing (UMGC). We identified whether the sequences contained the outcrosser SNP (A) or the selfer SNP (C) with the program ApE. We genotyped chloroplasts for most (> 85%) individuals within each taxon at each contact zone, except for the outcrosser at SM, for which we genotyped 40% of individuals (Supporting Information Table S1; total *n* = 360 individuals across contact zones).

### Analyses

#### Magnitude of admixture in nuclear and chloroplast genomes

For each individual, we estimated ancestry across the nuclear genome and derived an estimate of genome‐wide ancestry proportions using a hidden Markov model (HMM) designed for unphased sequencing data with low depth (Corbett‐Detig & Nielsen, 2017). Briefly, this HMM uses allele frequencies in reference panels of each ancestry type to determine the posterior probability that a given site is one of three ancestry genotypes, given read count data for the site and a recombination map. We used this method to quantify admixture in a whole‐genome‐sequencing dataset (Sianta *et al*., 2024), wherein reference panels for each taxon were constructed using multiple allopatric populations. We used the same reference panel individuals in this analysis, with the caveat that we subsampled the whole‐genome reference panels down to only genomic sites that were present in our GBS SNP VCF, were biallelic SNPs in the reference panel, had at least 10 of 30 reference panel individuals with data (including individuals of both taxa), and were separated by at least 200 bases. This resulted in 113 786 SNPs that were used as input in the HMM.

We performed the HMM analysis separately for individuals from each of the eight taxon/contact zone combinations (i.e. S6–outcrosser, S6–selfer, S22–outcrosser, S22–selfer, SAW–outcrosser, SAW–selfer, SM–outcrosser, SM–selfer), grouping individuals based on glasshouse phenotypic taxon ID, and using a uniform recombination map. The model included one admixture pulse, with the timing of admixture inferred by the HMM during the run. As in Sianta *et al*. (2024), we used the Baum‐Welch expectation–maximization algorithm to update the model input estimate for global admixture proportion. We reran the HMM until the output and input admixture proportions differed by < 0.001.

For each individual, the HMM outputs a posterior probability for each of three ancestry genotypes – homozygous heterospecific (HH), heterozygous con‐ and heterospecific (HC), and homozygous conspecific (CC) ancestry – per SNP. We calculated a genome‐wide admixture proportion, α, for each individual in two ways. First, we selected SNPs that had high confidence ancestry calls (posterior probability of most likely ancestry genotype > 0.9), hard‐called an ancestry genotype at each SNP, *i*, and calculated admixture proportion akin to the frequency of a heterospecific allele across high confidence SNPs, *n*_*hi.conf*_:

$$x_{i}\left\{\begin{array}{c} 0\;\text{if}\;\mathrm{CC}\\ 0.5\;\text{if}\;\mathrm{HC}\\ 1\;\text{if}\;\mathrm{HH} \end{array}\right.$$

$$\alpha =\frac{\sum_{i}^{n_{hi.conf}}x_{i}}{n_{hi.conf}}$$

Second, we calculated individual genome‐wide admixture proportions using all SNPs, regardless of whether an SNP had a high‐confidence (posterior probability >0.9) ancestry genotype. At each SNP, *i*, we calculated a weighted admixture score, $z_{i}$, with the admixture contribution of each genotype weighted by its posterior probability, w. We then averaged this weighted admixture score across all, *n*, SNPs:

$$z_{i}=0\left(w_{\mathrm{CC}}\right)+0.5\left(w_{\mathrm{HC}}\right)+1\left(w_{\mathrm{HH}}\right)$$

$$\alpha =\frac{\sum_{i}^{n}z_{i}}{n}$$

Individual‐level admixture proportions calculated using either only high confidence or all SNPs were highly correlated (Fig. S1, Table S2; average correlation coefficient of 0.94 for each taxon/contact zone combination), and we used proportions calculated from all SNPs in subsequent analyses unless otherwise noted.

To quantify the degree of chloroplast capture, we tallied the number of individuals of each taxon that had either the outcrosser or the selfer chloroplast genotype.

#### Modality of genomic ancestry and phenotypes within contact zones

We generated triangle plots to visualize the hybrid classes present within each contact zone (*sensu* Gompert *et al*., 2014; Shastry *et al*., 2021). Triangle plots show the proportion of the nuclear genome of a given ancestry on the x‐axis (here, presented as proportion outcrosser ancestry) and proportion of the genome that has heterozygous ancestry genotypes on the y‐axis. The distribution of points on a triangle with corners at (x, y: 0, 0), (1, 0), and (0.5, 1) displays the different hybrid classes in the population. For example, F1 hybrids would be clustered at the top triangle corner (0.5, 1), with an outcrosser ancestry proportion of 0.5 and all sites having heterozygous ancestries. F2s fall into the middle of the triangle and backcrosses fall along the edges of the triangle toward the purely parental types at the bottom corners. Because accurate ancestry calls are essential for interpretation of triangle plots, we used only sites that the HMM inferred with high confidence (i.e. posterior probability of the most likely genotype was > 0.9). An average of 94 300 sites across individuals passed the high confidence threshold.

We determined the modality of each contact zone based on genomic and phenotypic variation using Gaussian mixture models on all individuals within a contact zone. We used the Mclust() function of the R package mclust (v.6.0.1; Scrucca *et al*., 2023) to determine the optimal number of clusters and to assign individuals to clusters. For a given contact zone, we iteratively ran models over *K* = 1–9 and only accepted the next more‐complex model if it decreased BIC by at least 10. We chose delta‐BIC > 10 because it indicates very strong evidence for the more complex model (Raftery, 1995; Lorah & Womack, 2019; but see Nguyen & Nguyen, 2026, for discussion of BIC use and modifications in mixture model order selection). If contact zones are bimodal (i.e. primarily parental genotypes), we expect models to identify two clusters, one for each taxon. Whereas if contact zones are trimodal because of frequent hybridization, we expect models to identify two parental clusters plus a third cluster that represents early‐generation hybrids. Detection of more than two clusters may also occur if there is bimodality between taxa and population substructure within one or both taxa.

We performed Gaussian mixture models on the genomic dataset used in the HMM analyses, with an additional filter that removed SNPs with any missing data across all taxa and contact zones, resulting in a dataset of 5385 SNPs. We ran Gaussian mixture models on univariate phenotypic data for four phenotypes: petal size, herkogamy, protandry, and flowering time.

We found evidence of bimodality (*K* = 2) in genomic and phenotypic data in most cases. We detected *K* > 2 in the genomic data in two contact zones, but the additional mode was caused by substructure within the selfer rather than because of a high frequency of early‐generation hybrids. To examine whether substructure in the selfer at these two contact zones was caused by admixture (late‐generation introgression) or genetic structure unrelated to admixture, we tested whether clusters differed in admixture proportion using ANOVAs. We also visualized clusters using a genomic PCA, where we expected a more highly admixed selfer cluster to position closer to the outcrosser.

#### Testing for associations between admixture and phenotypes

An individual's admixture proportion may be associated with its phenotype for two reasons. Given that these floral phenotypes may be involved in reproductive isolation, certain phenotypes might prevent (or facilitate) hybridization/introgression. Alternatively, introgression may alter phenotypes such that individuals with high admixture proportions have similar trait values to the other taxon. We tested for an association between admixture proportions and phenotypes separately within each taxon using multiple regressions. We ran two sets of models per taxon: one in which we analyzed individuals from each contact zone separately and one in which we analyzed all individuals in the taxon together.

For each taxon and contact zone separately, we conducted a multiple regression of admixture proportion on petal size, herkogamy, protandry, and flowering time. In the three contact zones where the selfer is color polymorphic, we also included flower color as an independent variable (binary; pink vs white). To evaluate the potential for multicollinearity to affect multiple regression results, we checked correlations among all four quantitative variables within each taxon and contact zone and found low and largely insignificant trait correlations (mean ± SD: absolute *r* = 0.20 ± 0.06; Fig. S2). Multiple regressions were run with the lm() function in R stats package. We report model coefficients multiplied by 100 to aid in presentation in text and tables; thus, coefficients reflect the change in the percentage, instead of the proportion, of the genome that is admixed. We standardized each continuous predictor variable (petal size, herkogamy, protandry, and flowering time) before running models so that coefficient estimates were comparable across these predictors. We calculated the partial *R* ^2^ for each variable using the rsq.partial() function of the R rsq package. Partial *R* ^2^ values in this package are calculated as the difference between a full and reduced model; negative values indicate that the variable does not improve model fit nor uniquely explains variance and we have assigned negative partial *R* ^2^ values as zero. We assessed the significance of independent variables using Type II Sums of Squares with the ANOVA() function of the R car package (v.3.1–0; Fox & Weisberg, 2018). For the selfer in three contact zones (S6, S22, SAW), each of the three models was influenced by one outlier with very high protandry. We removed the three outliers (one per model), reran models, and present results of models without outliers. All variance inflation factors were < 5 (mean VIF = 1.4), indicating that variance explained by predictor variables was not substantially inflated by multicollinearity (vif() function, R car package; Table S3). We used the ggeffects R package (Lüdecke, 2018) to extract and visualize marginal means for flower color (using ggemmeans()) and predictions for petal size, herkogamy, protandry, and flowering time (using ggpredict()). For all models, we used the plot() function on the lm() model objects in R to visualize Q–Q and residual vs fitted values plots and check for violations of normality and homoscedasticity of residuals. All model validation plots passed visual inspection, with the caveat that there are roughly bimodal distributions in the selfer admixture proportions at SM and S22.

For each taxon across all contact zones combined, we modeled admixture proportions as a function of phenotypes in a mixed model framework, with contact zone as a random intercept and phenotypes as fixed effects. We standardized continuous variables before running the model, fit the model using the lmer() function of the R lme4 package (Bates *et al*., 2014), and assessed significance of phenotypes with the ANOVA() function, as above. We calculated partial *R* ^2^ values using the partR2() function of the partR2 package (Stoffel *et al*., 2021), which is designed for mixed models. We ran one model for the outcrosser, including all four contact zones and four phenotypes (petal size, herkogamy, protandry, and flowering time) and one model for the selfer, including all contact zones and five phenotypes (flower color, petal size, herkogamy, protandry, and flowering time). We extracted marginal effects for each phenotype, as above.

#### Testing for fine‐scale spatial patterns in ancestry and phenotypes within contact zones

We used two approaches to examine fine‐scale patterns of genetic and phenotypic structure within contact zones. First, we tested for isolation‐by‐distance within each taxon at each contact zone on two matrices: one composed of genetic distance and one composed of ancestry distance (see below). Second, we tested for a relationship between admixture proportion or phenotypic variation in relation to proximity to the heterospecific taxon.

To calculate the genetic distance, we removed all missing data from the SNP matrix used in the HMM analyses (*n* = 5385 SNPs remaining) and calculated Nei's genetic distance among individuals within each taxon/contact zone combination with the R adegenet package (Jombart, 2008; Jombart & Ahmed, 2011). Second, we created a matrix of ancestry distance, which describes the similarity in ancestry calls across the genome between individuals. A low ancestry distance represents two individuals that have similar ancestry calls across the genome, which could be caused by each individual having no admixture (i.e. all conspecific ancestry) or by each individual having admixture at the same genomic sites. We created this matrix by taking high confidence ancestry genotype calls from the HMM, removing all SNPs at which there were missing data (*n* = 1185 SNPs), and using Nei's genetic distance on the ancestry genotypes, as above. We calculated geographic distance among individuals within each taxon/contact zone combination using the dist() function in R. We performed separate Mantel tests for genetic and ancestry distance using the mantel() function in the R package vegan (Oksanen *et al*., 2025) to run Mantel tests with 1000 permutations.

Next, we tested whether there was a negative relationship between an individual's admixture proportion and its distance to the heterospecific taxon. For every individual, we calculated the average distance to the three nearest heterospecific individuals. For each taxon/contact zone combination, we fit a linear model of admixture proportion vs distance to heterospecifics. Because there was a clear curvilinear pattern for the selfer at the SM contact zone, we ran a model that included an additional quadratic term for distance to heterospecific. We used the ANOVA() function in the R stats package to test whether the model with the quadratic term was a better fit than the model without the quadratic term. We visualized Q–Q plots and residual vs fitted values plots to check for violations of normality and homoscedasticity of residuals; all models passed visual inspections.

## Results

### Higher admixture in northern than southern contact zones

**Fig. 2:**
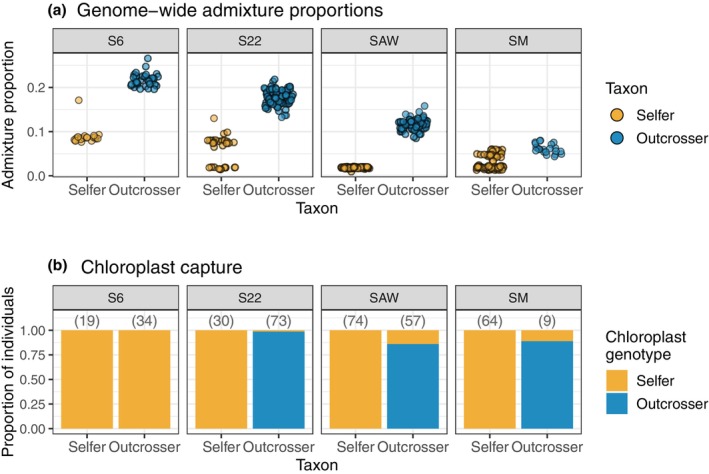
Introgression in both the nuclear and chloroplast genomes was asymmetric between *Clarkia xantiana* sister taxa and varied within and among contact zones. (a) Individual admixture proportions calculated from the hidden Markov model (HMM) using all sites. (b) The proportion of individuals in each taxon that had the selfer or outcrosser chloroplast genotype. Numbers above bars are the total number of individuals with genotyped chloroplasts. Of note is S6, where all outcrosser individuals had the selfer chloroplast genotype. S6, site 6; S22, site 22; SAW, Sawmill Road; SM, Squirrel Mountain.

Introgression was asymmetric, with more introgression into the outcrosser than the selfer (Fig. 2a). The admixture proportion in the outcrosser was highest in the northernmost contact zone, S6 (mean ± SE; 0.22 ± 0.003) and progressively decreased with latitude (S22 = 0.18 ± 0.002, SAW = 0.12 ± 0.002, SM = 0.06 ± 0.002). A similar pattern was observed in the selfer: admixture proportion was higher at the two northern sites (S6: 0.09 ± 0.004; S22: 0.06 ± 0.006) and lower at the southern sites (SM: 0.03 ± 0.002; SAW: 0.02 ± 0.0002). Unlike other populations, the ancestry distributions of the selfer at S22 and SM had two modes (Fig. 2a). Each population had a group of individuals with near‐zero admixture and a group with *c*. 5–10% admixture.

### Chloroplast capture in all outcrosser but no selfer populations

Similar to nuclear admixture proportion, chloroplast capture was also asymmetric (Fig. 2b). While we found no outcrosser‐derived chloroplasts in the selfer, every outcrosser population had at least one individual with a selfer‐derived chloroplast. In three of four contact zones, 1, 14, and 11% of outcrosser individuals had selfer chloroplasts (S22, SAW, and SM, respectively). Because only nine individuals were genotyped in SM, this estimate should be viewed with caution. In the northern‐most contact zone (S6), 100% of outcrossers (*n* = 34) had the selfer chloroplast.

### Contact zones were bimodal

**Fig. 3:**
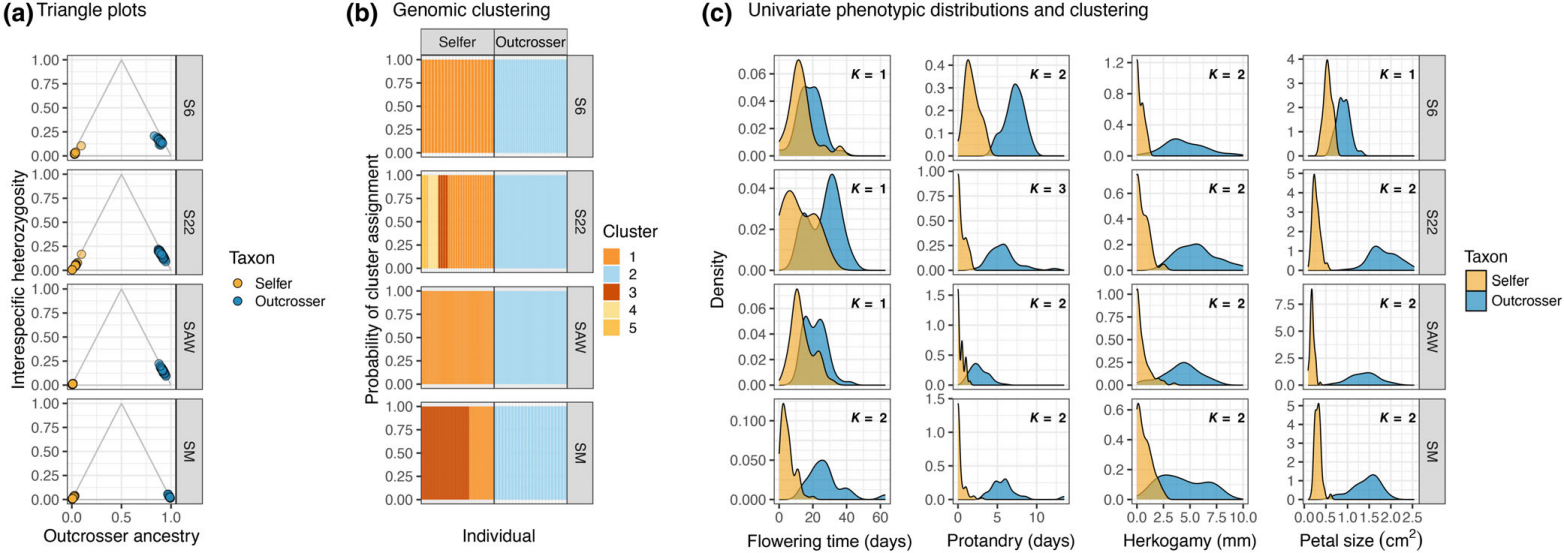
Genomic and phenotypic evidence supported bimodality of *Clarkia xantiana* contact zones. (a) Triangle plots show the proportion of an individual's genome that is derived from outcrosser ancestry (*x*‐axis) and the proportion of an individual's genome that is heterozygous for selfer and outcrosser ancestry (*y*‐axis). Pure parental‐like genotypes are expected in the bottom triangle corners, F1s in the top triangle corner, hybrid lineages (F2s, F3s, etc.) in the middle of the triangle, and backcrosses along the triangle edges. Our plots indicate that admixture is old and was followed by many generations of backcrossing. (b) Gaussian mixture models were independently performed within each contact zone to identify the number of clusters in the single‐nucleotide polymorphism data. The best‐fitting model identified *K* = 2 clusters for S6, *K* = 5 clusters for S22, *K* = 2 clusters for SAW, and *K* = 3 clusters for SM. (c) Univariate trait distributions of four phenotypes with the number of clusters, K, in each distribution identified by a Gaussian mixture model. We note flowering time is standardized by the earliest flowering individual in each contact zone (see Methods). S6, site 6; S22, site 22; SAW, Sawmill Road; SM, Squirrel Mountain.

Triangle plots and clustering models of genomic and phenotypic data suggest that all four contact zones are bimodal. We did not detect evidence of early generation hybrids in any contact zone. The observed admixture was a result of many generations of backcrossing (rather than recent hybridization), as individuals from all sites clustered in the bottom two corners and along the lower edges of the triangle plots (Fig. 3a). Gaussian mixture models, based on either genomic data (Fig. 3b) or phenotype data (Fig. 3c), grouped individuals of the two taxa within a contact zone into distinct clusters, indicating bimodality.

Despite evidence for bimodality in all contact zones, the best Gaussian mixture models did not always find *K* = 2 clusters for a given contact zone. In the best‐fitting models inferred from genomic data, the S22 and SM contact zones had *K* = 5 and *K* = 3 clusters, respectively, corresponding to within‐taxon clustering in the selfer (Fig. 3b). This substructure within the selfer appears to be influenced by variation in admixture. In S22, one cluster within the selfer had 2.9× higher admixture than the other three clusters (*F*_3,26_ = 70.0, *P* < 0.001; Fig. S3). In SM, one cluster had 2.7× higher admixture than another cluster (*F*_1,71_ = 640.0, *P* < 0.001; Fig. S3). PCAs within S22 and SM confirmed that these additional clusters were genetically distinct and that higher‐admixture clusters were more similar to the outcrosser (Fig. S3).

Likewise, the best‐fitting Gaussian mixture models did not find support for bimodality in four of the 16 univariate phenotype distributions (Fig. 3c). Flowering time had a unimodal distribution in three of the contact zones (S6, S22, and SAW). While petal size was strongly bimodal at S22, SAW, and SM, it had a unimodal distribution at the northernmost site, S6.

### Admixture was not associated with phenotypes in the outcrosser

There were no significant associations between admixture and phenotypes in the outcrosser at any of the four contact zones. However, at S22, a greater percentage admixture was nearly significantly associated with larger petal size (b (SE) = 0.43 (0.22); *F* = 3.93, *P* = 0.051; Table 1). Similarly, there were no associations between admixture and phenotypes in the model that included all outcrosser contact zones and phenotypes (Table 1).

**Table 1:** Phenotypes were associated with admixture in the *Clarkia xantiana* selfer taxon but not in the outcrosser taxon.

	Contact zone	Trait^1^	Selfer				Outcrosser			
			Estimate (SE)^2^	Partial *R* ^2^	Test statistic	*P*	Estimate (SE)^2^	Partial *R* ^2^	Test statistic	*P*
Contact zone‐specific models	S6	Flower color (pink–white)	NA	NA	NA	NA	NA	NA	NA	NA
		Flowering time	0.102 (0.168)	0	*F* = 0.37	0.554	−0.12 (0.264)	0	*F* = 0.21	0.652
		Protandry	−0.08 (0.143)	0	*F* = 0.31	0.583	0.289 (0.29)	0.06	*F* = 0.99	0.327
		Herkogamy	0.028 (0.111)	0	*F* = 0.06	0.806	−0.363 (0.271)	0	*F* = 1.79	0.191
		Petal size	0.106 (0.137)	0	*F* = 0.59	0.452	−0.178 (0.267)	0	*F* = 0.45	0.509
	S22	Flower color (pink–white)	2.269 (1.17)	0.13	*F* = 3.73	0.066	NA	NA	NA	NA
		Flowering time	0.567 (0.582)	0	*F* = 0.95	0.341	−0.296 (0.214)	0	*F* = 1.91	0.171
		Protandry	0.791 (0.612)	0	*F* = 1.66	0.210	0.241 (0.22)	0.06	*F* = 1.20	0.277
		Herkogamy	0.661 (0.494)	0	*F* = 1.79	0.195	0.216 (0.217)	0	*F* = 0.99	0.324
		Petal size	0.203 (0.543)	0	*F* = 0.14	0.712	0.427 (0.215)	0	*F* = 3.93	0.051
	SAW	Flower color (pink–white)	−0.013 (0.029)	0	*F* = 0.19	0.664	NA	NA	NA	NA
		Flowering time	0.013 (0.015)	0	*F* = 0.79	0.376	0.257 (0.184)	0	*F* = 1.95	0.168
		Protandry	−0.003 (0.015)	0	*F* = 0.03	0.865	−0.302 (0.176)	0.02	*F* = 2.95	0.091
		Herkogamy	−0.005 (0.014)	0	*F* = 0.12	0.734	0.23 (0.182)	0	*F* = 1.61	0.210
		Petal size	0.025 (0.014)	0	*F* = 3.03	0.086	0.006 (0.185)	0	*F* = 0.00	0.974
	SM	Flower color (pink–white)	**2.196 (0.34)** ^**3**^	**0.51**	***F* = 41.59**	**< 0.001**	NA	NA	NA	NA
		Flowering time	−0.198 (0.216)	0.02	*F* = 0.84	0.365	−0.428 (0.305)	0	*F* = 1.97	0.181
		Protandry	**0.686 (0.233)**	**0.18**	***F* = 8.70**	**0.005**	−0.19 (0.298)	0.05	*F* = 0.41	0.534
		Herkogamy	−0.205 (0.168)	0.04	*F* = 1.49	0.230	0.073 (0.282)	0	*F* = 0.07	0.800
		Petal size	**−0.373 (0.162)**	**0.12**	***F* = 5.26**	**0.027**	0.133 (0.289)	0	*F* = 0.21	0.653
Across contact zones model	All	Flower color (pink–white)	**1.20 (0.224)**	**0.048**	$\chi^{2}$ **= 28.87**	**< 0.001**	NA	NA	NA	NA
		Flowering time	**0.302 (0.121)**	**0.013**	$\chi^{2}$ **= 6.20**	**0.013**	−0.077 (0.123)	0	$\chi^{2}$ = 0.39	0.533
		Protandry	0.267 (0.138)	0.014	$\chi^{2}$ = 3.71	0.054	0.146 (0.170)	<0.001	$\chi^{2}$ = 0.74	0.390
		Herkogamy	−0.105 (0.106)	0.005	$\chi^{2}$ = 0.99	0.320	0.070 (0.122)	<0.001	$\chi^{2}$ = 0.33	0.564
		Petal size	0.116 (0.198)	0.007	$\chi^{2}$ = 0.35	0.557	0.213 (0.173)	<0.001	$\chi^{2}$ = 1.52	0.218

^1^ Models did not include the flower color term for taxon‐contact zone combinations in which there was no flower color polymorphism, indicated by NA.

^2^ Coefficient estimates for continuous variables (i.e. all but flower color) were standardized before running the model; thus, they represent the change in percent of the genome that is admixed as a function of one SD in the variable.

^3^ Bolded rows indicate significant model effects.

### Admixture was associated with some phenotypes in the selfer

Some contact zones showed associations between admixture and phenotype in the selfer when modeling each zone separately. At SM, greater percentage admixture was associated with pink (vs white) flowers (b (SE) = 2.20 (0.34); *F* = 41.59, *P* ≤ 0.001), higher protandry (b (SE) = 0.69 (0.23); *F* = 8.70, *P* ≤ 0.001), and smaller flowers (b (SE) = −0.37 (0.16); *F* = 5.26, *P* = 0.03) (Table 1). Higher admixture was also associated with pink (compared to white) flowers at S22, yet with a smaller effect size than at SM (pink flowers resulted in an increase in admixture proportion by 1.5× and 2.5× at S22 and SM, respectively). The strong association between admixture and flower color at S22 was not significant (b (SE) = 2.27 (1.17); *F* = 3.73, *P* = 0.07; Table 1) because there were few white‐flowered individuals (*n* = 10). By contrast, a lack of power cannot explain the lack of association between admixture and flower color at SAW (b (SE) = −0.013 (0.03); *F* = 0.19, *P* = 0.66, Table 1), which instead may be due to very low variation in admixture at this contact zone. There were no associations between admixture and phenotypes in the selfer at S6 and SAW.

**Fig. 4:**
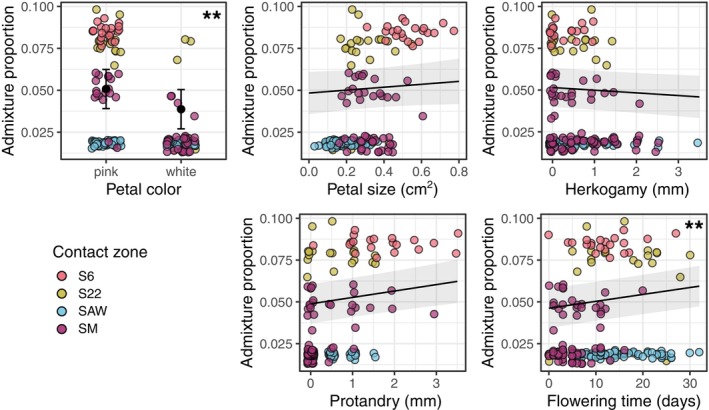
Multiple floral phenotypes were associated with admixture in the *Clarkia xantiana* selfer taxon. Presented are raw data (colored points) and predicted marginal effects from the across‐contact zones mixed model (run without standardizing continuous variables, for visualization). Marginal effects show mean predicted values (black points or lines) with one SE (error bars or grey ribbons). Asterisks in top right corner indicate significance of model effects. *< 0.05, **< 0.001. S6, site 6; S22, site 22; SAW, Sawmill Road; SM, Squirrel Mountain.

In our combined analysis across all sites, we also found associations between admixture and some phenotypes (Table 1, Fig. 4). Specifically, higher admixture was significantly associated with pink flowers (1.3× greater than with white flowers; b (SE) = 1.20 (0.22); *P* ≤ 0.001), later flowering time (b (SE) = 0.30 (0.12); *P* = 0.013), and nearly significantly associated with greater protandry (b (SE) = 0.26 (0.14); *P* = 0.054; Table 1, Fig. 4). By contrast, there was no significant association between admixture proportion and petal size or herkogamy (Table 1, Fig. 4).

### Spatial autocorrelation of genetic diversity but not shared ancestry

While there was a stronger signal of spatial autocorrelation in genetic distance in the selfer (average *r* = 0.38) than the outcrosser (average *r* = 0.18; Fig. S4, Table S4), neither taxon showed strong signals in spatial autocorrelation in ancestry (Fig. S4, Table S5).

### Admixture was often higher in the selfer when spatially proximal to the outcrosser

**Fig. 5:**
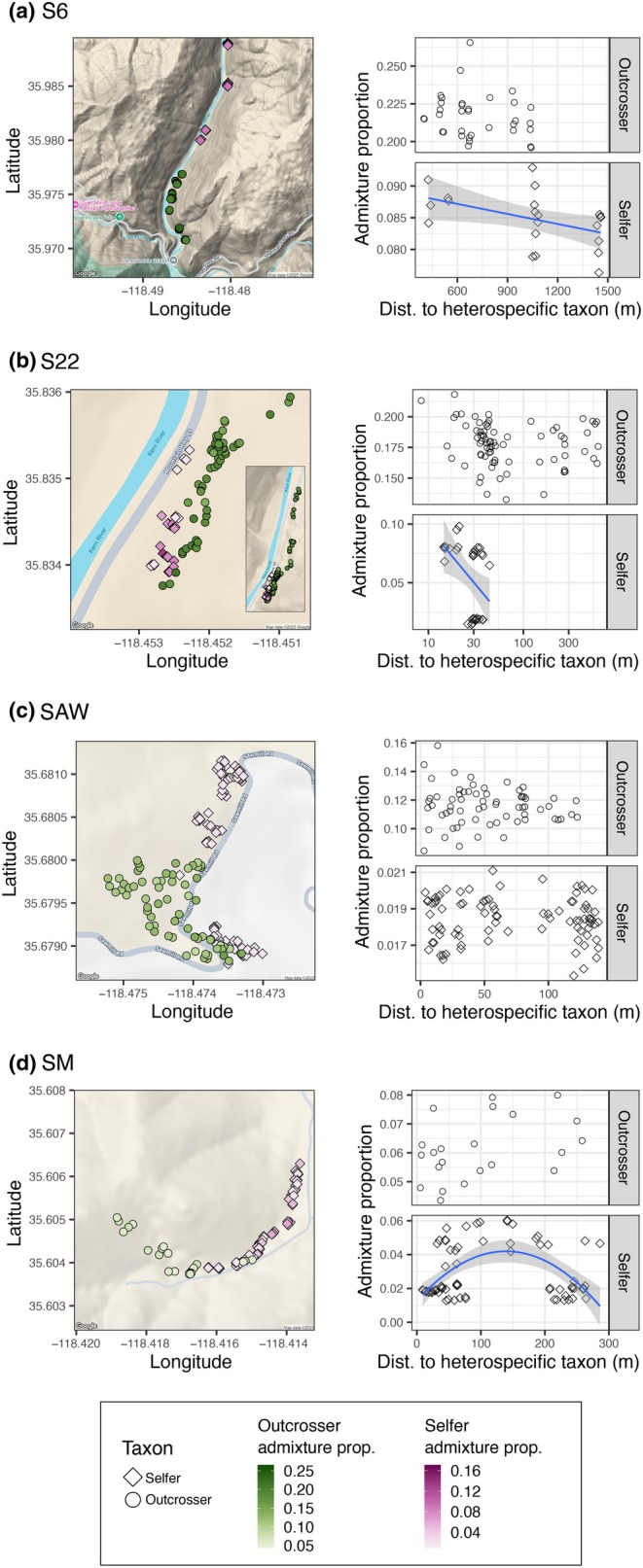
Individual‐level admixture proportions were explained by distance to nearest heterospecific neighbors in the selfer in some contact zones, but not in the outcrosser. Rows correspond to each contact zone: (a) Site 6 (S6), (b) Site 22 (S22), (c) Sawmill Road (SAW), (d) Squirrel Mountain (SM). Maps highlight the admixture proportions of outcrosser (green circles) and selfer (pink diamonds) *Clarkia xantiana* individuals on the landscape. The color scale is consistent across contact zones. To the right of each map are the individual admixture proportions of a focal taxon (outcrosser, top row; selfer, bottom row) as a function of average distance to the nearest three heterospecific individuals. In the three selfers with a significant relationship between the two variables, the regression line and 95% CI are plotted. Note in (b) the *x*‐axis for S22 is on a log_10_ scale to aid in visualization.

Individual admixture proportions were predicted by distance to the heterospecific taxon in the selfer at three of the four contact zones (Table 2, Fig. 5). Selfer individuals with smaller geographic distances to the outcrosser had higher admixture proportions at S6 (*F* = 5.34, *P* = 0.032) and S22 (*F* = 5.73, *P* = 0.024). There was a clear curvilinear pattern between admixture proportion and distance to the heterospecific taxon in the selfer at SM (Fig. 5d) and adding a quadratic term to the linear model significantly improved model fit (*F* = 21.20, *P* < 0.001). There was no significant relationship between admixture proportion and distance to the heterospecific taxon in the outcrosser at any contact zone (Table 2).

**Table 2:** Distance to the heterospecific taxon predicted admixture proportion in the selfer at three *Clarkia xantiana* contact zones, but not in the outcrosser.

Contact zone	Selfer		Outcrosser	
	*F*	*P*	*F*	*P*
S6	**5.34** ^**2**^	**0.032**	1.27	0.268
S22	**5.72**	**0.024**	7.76e^−4^	0.978
SAW	0.26	0.612	0.66	0.417
SM	0.15	0.701	3.77	0.066
SM (with quadratic term)^1^	**21.19**	**< 0.001**	NA	NA

^1^ In the selfer at contact zone SM, we present results for both the regular linear model (admixture ~ distance) and the linear model with a quadratic term (admixture ~ distance + distance^2^). The model with the quadratic term is a significantly better fit and we report the Sums of Squares for the quadratic term.

^2^ Bolded rows indicate a significant effect of distance to the heterospecific taxon on admixture proportion.

## Discussion

The outcome of secondary contact between recently diverged lineages reflects the push and pull of allopatric divergence, sympatric hybridization, and selection on admixed individuals. In plants, mating system divergence is often associated with speciation, but the mechanisms that influence the direction and magnitude of introgression are not fully understood. Here, we find no evidence for early‐generation hybrids between recently diverged *Clarkia* species despite little intrinsic postzygotic isolation. Yet we observed asymmetric introgression (from selfer to outcrosser) that varied in magnitude across contact zones: northern contact zones had higher introgression than southern ones. In addition, we observed chloroplast capture in the outcrosser (but never the selfer), ranging among populations from complete capture to just a few individuals. Within contact zones, variation in admixture was sometimes associated with phenotypes and spatial proximity to heterospecifics in the selfer, but not the outcrosser. Taken together, these results indicate that mating system has had a strong influence on the direction and spatial structure of introgression across contact zones.

### Contact zones are bimodal with no early‐generation hybrids

The four contact zones sampled in this study are bimodal contact zones with no evidence for early generation hybrids based on nuclear genomic and phenotypic data. This result is surprising given that the taxa are recently diverged (56–65 ky) and have only modest intrinsic postzygotic reproductive isolation (Pettengill & Moeller, 2012b; Briscoe Runquist *et al*., 2014; Sianta *et al*., 2024). Instead, the strong prezygotic isolation in this system must play a central role in limiting hybridization and maintaining taxon boundaries (Harrison & Bogdanowicz, 1997; Jiggins & Mallet, 2000). Phenological isolation, pollinator preference, and conspecific pollen precedence all contribute to prezygotic isolation in sympatry (Briscoe Runquist *et al*., 2014). Prezygotic isolation is not complete, however, and may be complemented by extrinsic selection against hybrids. The habitat and mating system divergence between taxa suggest that hybrids have low fitness in parental microhabitats and/or in mating success, and we are currently quantifying hybrid fitness in the field.

The only trait for which there was little evidence of bimodality was flowering time. This result suggests that alleles influencing flowering time have adaptively introgressed, have minimal effects on fitness, or that temporal variation in selection has slowed their elimination. Given that phenological isolation is a strong barrier in the field (RI = 0.79–0.96; Briscoe Runquist *et al*., 2014), it is surprising that there were not distinct flowering time distributions at most contact zones. One possibility is that our metric of flowering time may not be reflective of overall overlap in flowering time. Even if peak flowering times are similar between taxa, variation in flowering duration may result in phenological isolation (e.g. Farnitano *et al*., 2025). A second possibility is that glasshouse conditions do not closely simulate field conditions and cause greater overlap in flowering time. Plasticity in flowering time is common (Anderson *et al*., 2012; Jordan *et al*., 2015; Ramirez‐Parada *et al*., 2023) and can affect the degree of phenological isolation in a variety of systems (Dittmar & Schemske, 2017; Sianta & Kay, 2021).

### Mechanisms of asymmetric introgression

Introgression between the taxa was asymmetric across all four contact zones, with more introgression into the outcrosser than into the selfer. We found the same asymmetry in a prior study that used whole‐genome sequences but sampled considerably fewer individuals (Sianta *et al*., 2024). Asymmetric introgression from outcrossers to selfers has been noted in multiple systems while evidence of the reverse is limited (Sweigart & Willis, 2003; Ruhsam *et al*., 2011; Pettengill & Moeller, 2012b; Brandvain *et al*., 2014; Rifkin *et al*., 2019; Sianta *et al*., 2024). Evidence of chloroplast capture suggests that hybrids primarily form on the selfer with subsequent backcrossing to the outcrosser. Because pollinators primarily visit outcrossers (Briscoe Runquist *et al*., 2017) and outcrossers typically far outnumber selfers in contact zones, the probability of pollen flow from outcrosser to selfer is considerably higher. While chloroplast capture is seen in a variety of systems (Rieseberg & Soltis, 1991; Bock *et al*., 2014), it is typically studied at the phylogenetic level, with one to few samples per taxon. Ours is one of relatively few studies (e.g. Whittemore & Schaal, 1991; Petit *et al*., 2002; Palme *et al*., 2004; Farnitano *et al*., 2025) that have investigated chloroplast capture at a finer taxonomic level – sampling densely among individuals and populations.

We found low levels of chloroplast capture at three contact zones (1.4–14%) but complete capture at the northernmost contact zone, S6, which also had the highest nuclear admixture. This variation among contact zones could reflect different demographic histories associated with secondary contact and hybridization. Prior evidence suggests that Holocene range expansion in both taxa has occurred to more northern and high elevation areas (Pettengill & Moeller, 2012a). One possibility is that S6, the northernmost contact zone site, is recently formed and that fixation resulted from a strong bottleneck. A second possibility is that reproductive isolation is weak because there has been less time for selection to cause stronger barriers in this area of sympatry. Site 6 is particularly unusual in floral morphology: outcrosser petals are unusually small but flowers have protandry and herkogamy (Moeller, 2006). Landscape‐level variation in chloroplast capture could also reflect adaptive introgression. If the cytoplasmic genome contributes to the higher photosynthetic rates and faster development characteristic of the selfer (Mazer *et al*., 2010; Burnette & Eckhart, 2021), selection may favor outcrossers with a selfer chloroplast at some sites. Our results in S6 are similar to those of Farnitano *et al*. (2025) who recently found complete chloroplast capture by a *Mimulus* outcrosser in two contact zones with a sister selfer species. Future work could distinguish among these hypotheses with field experiments that involve cytoplasmic introgression lines.

### Admixture is associated with specific phenotypes in the selfer, but not the outcrosser

In the selfer, we found evidence across models that flower color (pink vs white), protandry, and flowering time predicted variation in admixture. An association between admixture and phenotypes could arise in two ways: (1) individuals with certain phenotypic values are more likely to hybridize with heterospecifics and/or hybrids or (2) introgression results in individuals with heterospecific‐like trait values. For example, we found that selfer individuals with later flowering time and higher protandry had higher admixture proportions. Early flowering and low protandry could prevent hybridization and introgression due to temporal isolation and preemptive selfing, respectively. Alternatively, introgression of outcrosser alleles at genomic regions underlying flowering time and protandry into admixed selfer individuals could result in a similar pattern. We are currently conducting experiments involving genetic lines that shuffle trait combinations to test whether flower color, protandry, and flowering time affect pollinator visitation and hybridization. Additionally, comparisons of ancestry in genomic regions underlying these traits relative to a genome‐wide null expectation could help distinguish these two alternative explanations.

We suspect that flower color has a causal effect on hybridization and introgression, mediated by differential pollinator visitation to flower color morphs. The pink morph is common across the range of the selfer (i.e. not only found in sympatry) and most populations (98% of 46 populations with at least 10 individuals in 2001) are pink or polymorphic (M.A. Geber and V.M. Eckhart, unpublished data). Moreover, field experiments with recombinant inbred lines found that pink genotypes had significantly more bee visitors than white genotypes (Kern *et al*., unpublished). These observations suggest that pink flowers in sympatric selfer populations are not likely caused by introgression from the outcrosser. While it is well known that flower color differences between species can influence hybridization (e.g. Schemske & Bradshaw Jr., 1999; Hopkins & Rausher, 2012) our study is unusual in showing that flower color variation *within* populations is associated with admixture across the genome.

### Mating system predicts fine‐scale spatial variation in admixture

Much like phenotypic variation, admixture was only structured at fine spatial scales in the selfer, not in the outcrosser. In two contact zones, we found a negative relationship between admixture proportion and distance to heterospecifics, suggesting that hybrids are more likely to form where taxa are in close proximity. That we did not observe this pattern in the outcrosser is not surprising given that we detected no early‐generation hybrids and that pollen dispersal is high in outcrossing populations and low in primarily selfing populations. In the selfer, pollen flow is limited because pollinator visitation is uncommon (Moeller, 2006; Briscoe Runquist *et al*., 2014). In the outcrosser, pollen may disperse 70 m or more in a single generation (Kern *et al*., 2023). Thus, pollen flow could homogenize admixed ancestry in an outcrosser population within a few generations. Indeed, we see little signal of spatial autocorrelation in genetic diversity or admixed ancestry in any of the outcrosser populations.

Spatial patterns in admixture may also be a function of selection caused by environmental variation. Given the differences in physiology and life history, it is likely that the spatial partitioning of taxa within contact zones is driven by microhabitat variation. Selfers with higher admixture proportions may be restricted to more outcrosser‐like microhabitats that are at the ecotone between two adjacent microhabitats. For example, we found a negative relationship between admixture and distance to heterospecifics at S6 and S22. If the strength of selection against admixed ancestry increases with distance from the ecotone, this could be a mechanism that explains spatial patterns of introgression. Likewise, selection across a fine‐scale mosaic of microhabitats within the selfer population at SM could result in the curvilinear relationship between admixture and distance to heterospecifics at SM. Ongoing work examines this possibility using field transplant experiments.

### Conclusions

Despite very recent divergence, evidence of historical introgression, and dense sampling, our results are surprising in uncovering no early generation hybrids. At a geographic scale, admixture was highest (and chloroplast capture complete) at the latitudinal and elevational extreme of the zone of sympatry. At a fine scale, we found that the mating system mediated the direction and spatial structure of admixture. In particular, admixture was associated with phenotypes that promote outcrossing and spatial position in the selfer, but not the outcrosser. While past work has shown that flower color divergence can be pivotal for reproductive isolation, our work is unusual in revealing how naturally occurring variation within species may modulate genome‐wide admixture. Taken together, mating system evolution can rapidly isolate selfing derivatives, but the early stages of speciation involve substantial introgression that is mediated by the ecological and phenotypic variation within contact zones.

## Competing interests

None declared.

## Author contributions

SAS, DM, and YB designed the study; DM conducted field work; BK and DM collected common garden data and tissue samples; AS and SAS collected cpDNA data; SAS, BK, YB, and DM designed and/or performed analyses; SAS led and DM and YB contributed substantially to the writing of the manuscript; other authors contributed to editing.

## Disclaimer

The New Phytologist Foundation remains neutral with regard to jurisdictional claims in maps and in any institutional affiliations.

## Supporting information


**Fig. S1** Correlations between estimates of HMM‐derived admixture proportions of *Clarkia xantiana* individuals calculated with all sites or high‐confidence sites.
**Fig. S2** Correlations among mating system phenotypes in *Clarkia xantiana* taxa.
**Fig. S3** Characterization of within‐selfer genomic clusters at the S22 and SM *Clarkia xantiana* contact zones.
**Fig. S4** Observed and permuted estimates of isolation by genetic distance and isolation by ancestry distance in *Clarkia xantiana* taxa.
**Table S1** Sample size of *Clarkia xantiana* individuals per contact zone and taxon that had chloroplasts successfully genotyped.
**Table S2** Correlation coefficients between estimates of HMM‐derived individual‐level admixture proportions calculated with all sites or high‐confidence sites for the *Clarkia xantiana* selfer and outcrosser taxa.
**Table S3** Variance inflation factors calculated from within‐contact zone multiple regression models for each *Clarkia xantiana* taxon.
**Table S4** Correlations between geographic distance and genetic distance matrices across *Clarkia xantiana* taxa and contact zones.
**Table S5** Correlations between geographic distance and ancestry distance matrices across *Clarkia xantiana* taxa and contact zones.
Please note: Wiley is not responsible for the content or functionality of any Supporting Information supplied by the authors. Any queries (other than missing material) should be directed to the *New Phytologist* Central Office.

## Data Availability

Raw GBS sequence data were deposited in NCBI SRA (BioProject PRJNA1257434). Processed genomic data and data analysis code were deposited in figshare (doi: 10.6084/m9.figshare.29132639.v1).
