# Supplementary material for: Introgression despite minimal hybridization: mating system modulates phenotypic associations with introgression in Clarkia
Source: New Phytol. 2026 Mar 27;250(4):2619–33. doi: 10.1111/nph.71113 (PMC13103427; doi:10.1111/nph.71113)
Supplement: Supplementary file 1 — Fig. S1 Correlations between estimates of HMM‐derived admixture proportions of Clarkia xantiana individuals calculated with all sites or high‐confidence sites. Fig. S2 Correlations among mating system phenotypes in Clarkia xantiana taxa. Fig. S3 Characterization of within‐selfer genomic clusters at the S22 and SM Clarkia xantiana contact zones. Fig. S4 Observed and permuted estimates of isolation by genetic distance and isolation by ancestry distance in Clarkia xantiana taxa. Table S1 Sample size of Clarkia xantiana individuals per contact zone and taxon that had chloroplasts successfully genotyped. Table S2 Correlation coefficients between estimates of HMM‐derived individual‐level admixture proportions calculated with all sites or high‐confidence sites for the Clarkia xantiana selfer and outcrosser taxa. Table S3 Variance inflation factors calculated from within‐contact zone multiple regression models for each Clarkia xantiana taxon. Table S4 Correlations between geographic distance and genetic distance matrices across Clarkia xantiana taxa and contact zones. Table S5 Correlations between geographic distance and ancestry distance matrices across Clarkia xantiana taxa and contact zones. Please note: Wiley is not responsible for the content or functionality of any Supporting Information supplied by the authors. Any queries (other than missing material) should be directed to the New Phytologist Central Office. [file NPH-250-2619-s001.pdf]

New Phytologist Supporting Information

Article title: Introgression despite minimal hybridization: mating system modulates phenotypic associations with introgression in *Clarkia*

Authors: Shelley A. Sianta, Brooke R. Kern, Amal Suri, Yaniv Brandvain, David A. Moeller

Article acceptance date: 6 February 2026

Supplementary Figures

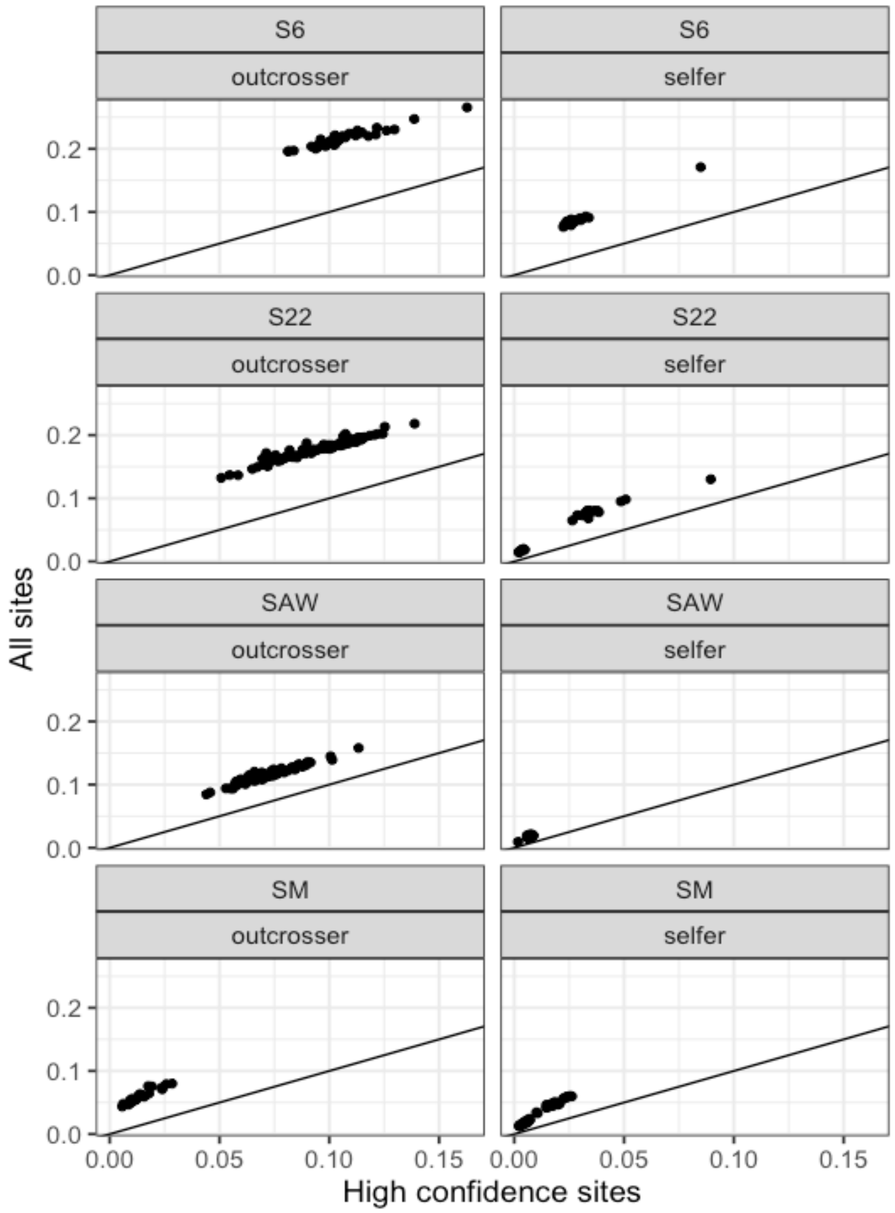

Figure S1: Calculations of HMM-derived admixture proportions of *Clarkia xantiana* individuals using only high-confidence sites (posterior probability of ancestry genotype > 0.9; x-axis) and all sites (y-axis) were highly correlated. Solid line represents a 1:1 relationship.

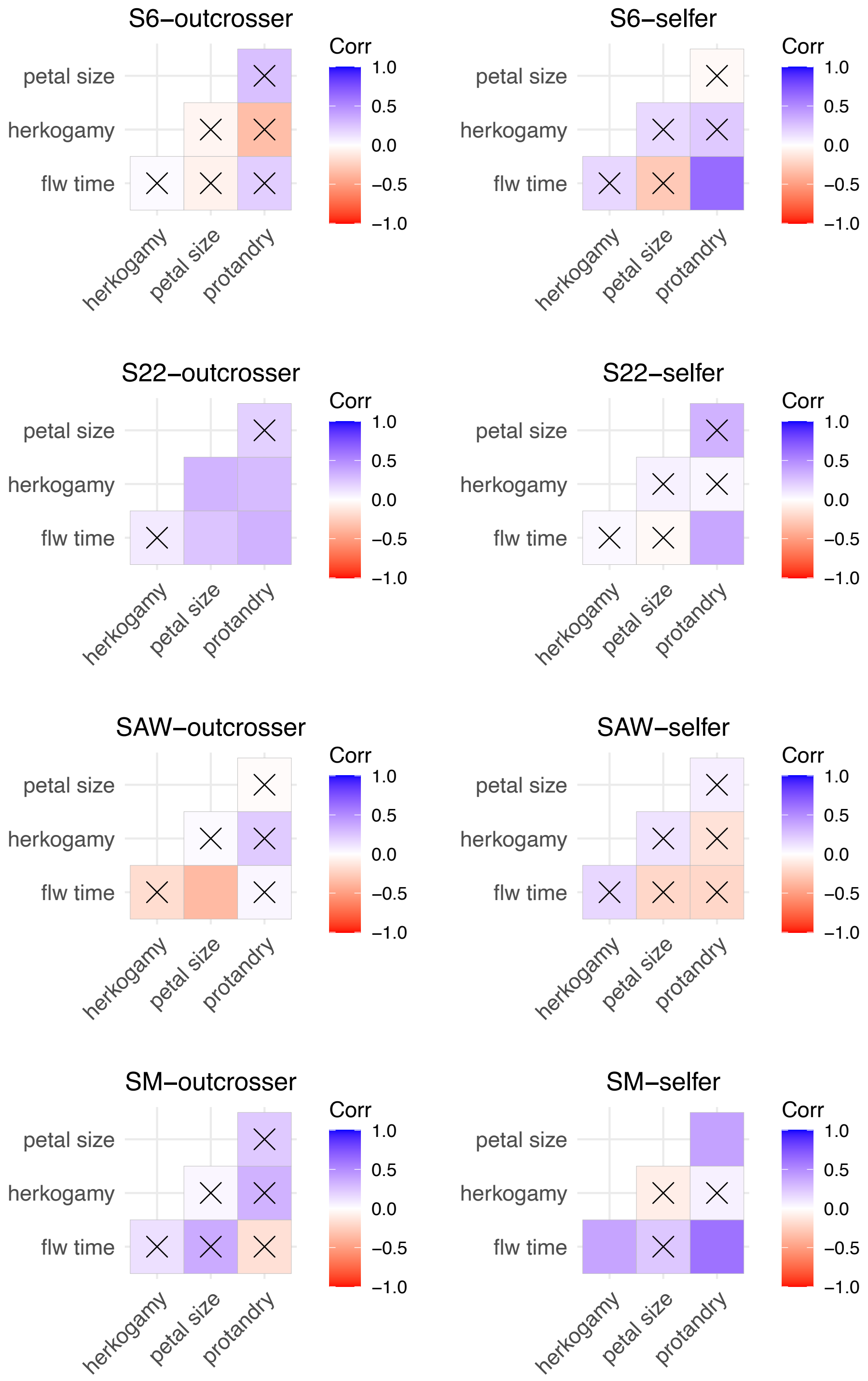

Figure S2: Reproductive phenotypes were weakly correlated with one another. For each *Clarkia xantiana* taxon-contact zone combination, we show Pearson correlations among the four continuous traits used in multiple regression analyses. Color of cells corresponds to the value of the correlation coefficient and cells that are covered with an "X" have non-significant correlations.

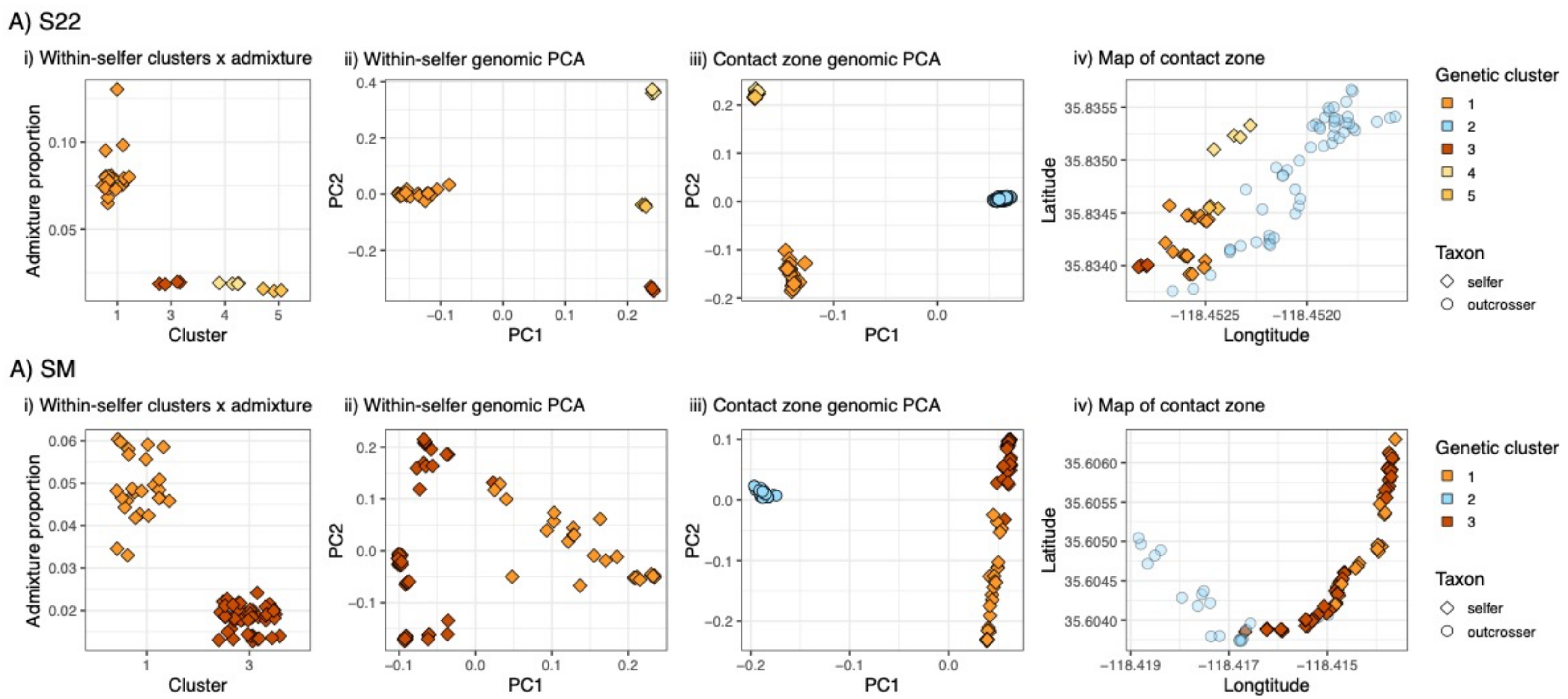

Figure S3: Within-selfer genomic clusters at S22 and SM differed in admixture proportion, were genetically distinct, and occupied separate parts of the contact zone. Genomic Gaussian mixture models identified within-selfer clusters at the S22 (A) and SM (B) *Clarkia xantiana* contact zones. (i) Within-selfer clusters at each site significantly differed in admixture proportions. (ii) Genomic PCAs of selfer individuals showed that individuals assigned to different genomic clusters were genetically distinct. (iii) The within-selfer clusters with higher admixture proportions were also more similar to the outcrosser (blue circles) on PC1. (iv) The within-selfer clusters occupy distinct geographic locations.

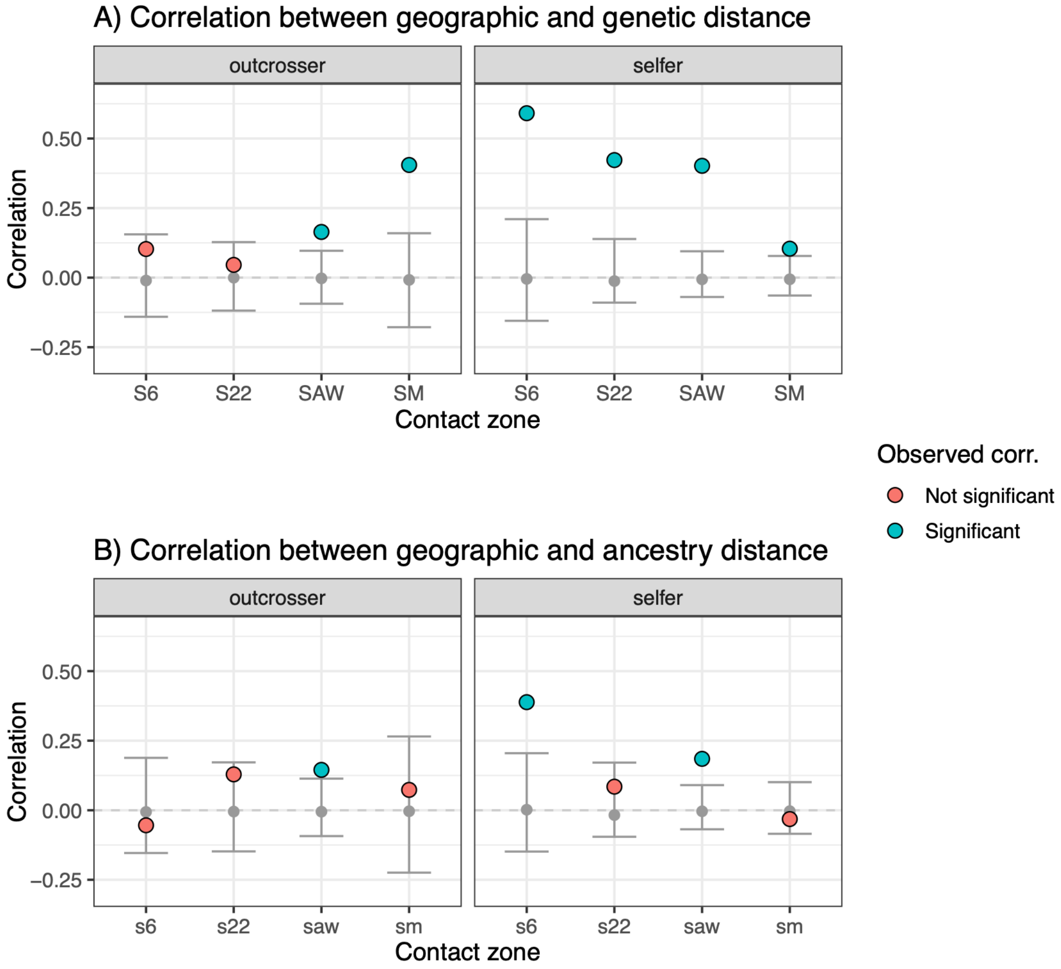

Figure S4: Shared ancestry across the genome had less spatial autocorrelation than genetic diversity in *Clarkia xantiana* outcrosser and selfer taxa. (A) Mantel test correlations between matrices of geographic distance and genetic distance, based on a matrix of genotype type calls at 5,385 SNPs. Colored points indicate observed correlation coefficients, with colors indicating significance based on permutation tests (grey points and bars, representing median and 95% CI values, respectively). (B) Mantel test correlations between matrices of geographic distance and distance in ancestry calls across the genome, based on a matrix of ancestry calls at 1,185 SNPs.

Supplementary Tables

Contact zone	Taxon	Sample size	
		All individuals	Individuals with genotyped chloroplasts
S6	Selfer	21	19
	Outcrosser	34	34
S22	Selfer	30	30
	Outcrosser	77	73
SAW	Selfer	89	76
	Outcrosser	68	60
SM	Selfer	73	64
	Outcrosser	22	9

Table S1: Sample size of *Clarkia xantiana* individuals per contact zone and taxon that had chloroplasts successfully genotyped. Note, the total number of individuals reflects the number of individuals that made it through the GBS filtering protocols.

Contact zone	Selfer	Outcrosser
S6	0.99	0.96
S22	0.97	0.97
SAW	0.77	0.97
SM	0.99	0.94

Table S2: Correlation coefficients between an individual's admixture proportion when calculated from the HMM output using all sites or only high confidence sites (posterior distribution > 0.9) for the *Clarkia xantiana* selfer and outcrosser taxa.

Taxon	Contact zone	Herkogamy	Protandry	Flowering time	Petal area	Flower color
Selfer	S6	1.08	1.87	2.30	1.44	NA
	S22	1.13	1.73	1.57	1.37	1.52
	SAW	1.12	1.19	1.14	1.10	1.16
	SM	1.29	2.34	2.19	1.22	1.31
Outcrosser	S6	1.14	1.30	1.08	1.10	NA
	S22	1.19	1.22	1.18	1.18	NA
	SAW	1.09	1.06	1.20	1.15	NA
	SM	1.19	1.33	1.32	1.30	NA

Table S3: Variance inflation factors calculated from within-contact zone admixture-by-phenotype multiple regression model outputs for each *Clarkia xantiana* taxon. All variance inflation factors were less than 5, which indicates that multicollinearity is not a problem the multiple regression models.

Taxon	Contact Zone	Observed correlation	95% CI (lower)	95% CI (upper)	p-value
Selfer	S6	0.591	-0.158	0.189	0.001
	S22	0.422	-0.090	0.140	0.001
	SAW	0.402	-0.068	0.088	0.001
	SM	0.104	-0.065	0.083	0.005
Outcrosser	S6	0.103	-0.142	0.153	0.091
	S22	0.046	-0.117	0.118	0.234
	SAW	0.164	-0.096	0.095	0.001
	SM	0.405	-0.171	0.169	0.001

Table S4: Correlations between geographic distance and genetic distance matrices across *Clarkia xantiana* taxa and contact zones. Mantel test permutations were used to derive 95% confidence intervals and p-values.

Taxon	Contact Zone	Observed correlation	95% CI (lower)	95% CI (upper)	p-value
Selfer	S6	0.388	-0.149	0.205	0.001
	S22	0.085	-0.092	0.178	0.151
	SAW	0.185	-0.066	0.081	0.001
	SM	-0.032	-0.078	0.097	0.747
Outcrosser	S6	-0.206	-0.231	0.326	0.948
	S22	0.129	-0.143	0.175	0.068
	SAW	0.145	-0.098	0.110	0.006
	SM	0.073	-0.217	0.254	0.262

Table S5: Correlations between geographic distance and ancestry distance matrices across *Clarkia xantiana* taxa and contact zones. Mantel test permutations were used to derive 95% confidence intervals and p-values.
